# COVIDHunter: COVID-19 Pandemic Wave Prediction and Mitigation via Seasonality Aware Modeling

**DOI:** 10.3389/fpubh.2022.877621

**Published:** 2022-06-17

**Authors:** Mohammed Alser, Jeremie S. Kim, Nour Almadhoun Alserr, Stefan W. Tell, Onur Mutlu

**Affiliations:** Department of Information Technology and Electrical Engineering (D-ITET), ETH Zurich, Zurich, Switzerland

**Keywords:** epidemiological modeling, COVID-19 outbreak simulation, seasonal epidemic, outbreak prevention and control, vaccination

## Abstract

Early detection and isolation of COVID-19 patients are essential for successful implementation of mitigation strategies and eventually curbing the disease spread. With a limited number of daily COVID-19 tests performed in every country, simulating the COVID-19 spread along with the potential effect of each mitigation strategy currently remains one of the most effective ways in managing the healthcare system and guiding policy-makers. We introduce COVIDHunter, a flexible and accurate COVID-19 outbreak simulation model that evaluates the current mitigation measures that are applied to a region, predicts COVID-19 statistics (the daily number of cases, hospitalizations, and deaths), and provides suggestions on what strength the upcoming mitigation measure should be. The key idea of COVIDHunter is to quantify the spread of COVID-19 in a geographical region by simulating the average number of new infections caused by an infected person considering the effect of external factors, such as environmental conditions (e.g., climate, temperature, humidity), different variants of concern, vaccination rate, and mitigation measures. Using Switzerland as a case study, COVIDHunter estimates that we are experiencing a deadly new wave that will peak on 26 January 2022, which is very similar in numbers to the wave we had in February 2020. The policy-makers have only one choice that is to increase the strength of the currently applied mitigation measures for 30 days. Unlike existing models, the COVIDHunter model accurately monitors and predicts the daily number of cases, hospitalizations, and deaths due to COVID-19. Our model is flexible to configure and simple to modify for modeling different scenarios under different environmental conditions and mitigation measures. We release the source code of the COVIDHunter implementation at https://github.com/CMU-SAFARI/COVIDHunter and show how to flexibly configure our model for any scenario and easily extend it for different measures and conditions than we account for.

## Introduction

*Coronavirus disease 2019* (COVID-19) is caused by SARS-CoV-2 virus, which has rapidly spread to nearly every corner of the globe and has been declared a pandemic in March 2020 by the World Health Organization (WHO) (1). As of November 2021, only about 40% of the entire world population is fully vaccinated and their protection wanes after a few months (2). Until an effective drug or vaccination is made widely available to everyone, early detection and isolation of COVID-19 patients remain essential for effectively curbing the disease spread (3). Regardless of the availability and affordability of COVID-19 testing, it is still extremely challenging to detect and isolate COVID-19 infections at early stages (4, 5). Simulating the spread of COVID-19 has the potential to mitigate such challenges, help to better manage the healthcare system, and provide guidance to policy-makers on the effectiveness of various (current, planned, or discussed) mitigation measures. To this end, many COVID-19 simulation models are proposed (6–10), some of which are announced to *assist* in decision-making for policy-makers in countries such as the United Kingdom [ICL (9)], United States [IHME (10)], and Switzerland [IBZ (11)].

These models tend to follow one of two key approaches. The first approach evaluates the current actual epidemiological situation by accounting for reporting delays and under-reporting (uncertainty) due to inefficiencies such as a low number of COVID-19 tests. This approach is taken by the IBZ (11), LSHTM (7), and (8) models and is *not* mainly used for prediction purposes as it reflects the epidemiological situation with about 2 weeks of time delay (due to its dependence on observed COVID-19 reports). The IBZ model (11) estimates the daily reproduction number, *R*, of SARS-CoV-2 from observed COVID-19 incidence time series data after accounting for reporting delays and under-reporting using the numbers of confirmed hospitalizations and deaths. The *R* number describes how a pathogen spreads in a particular population by quantifying the average number of new infections caused by each infected person at a given point in time (12). The LSHTM model (7) adjusts the daily number of observed COVID-19 cases by accounting for under-reporting (uncertainty) using both deaths-to-cases ratio estimates and correcting for delays between case confirmation (i.e., laboratory-confirmed infection) to death.

The second approach evaluates the current epidemiological situation and predicts the future epidemiological situation by simulating the COVID-19 outbreak and considering the effects of mitigation measures. This approach, taken by ICL (9) and IHME (10) models, usually suffers from two main drawbacks. The first drawback is that they require a large number of country-specific assumptions and input parameters (e.g., mobility rates, age- and country-specific data on demographics, patterns of social contact, and hospital availability) as it does not rely on the observed (laboratory-confirmed) number of cases for each region in simulation. For example, ICL (9) model requires input parameters such as the daily number of confirmed deaths, IFR, mobility rates from Google, age- and country-specific data on demographics, patterns of social contact, and hospital availability. This model makes three key assumptions: (1) age-specific IFRs observed in China and Europe are the same across every country, (2) the number of confirmed deaths is equal to the true number of COVID-19 deaths, and (3) the change in transmission rates is a function of average mobility trends. Another example is the IHME (10) model, which requires input parameters such as testing rates, mobility, social distancing policies, population density, altitude, smoking rates, self-reported contacts, and mask use. This model makes two key assumptions: (1) the infection fatality rate (IFR), which indicates the rate of people that die from the infection is taken using data from the Diamond Princess Cruise ship and New Zealand and (2) the decreasing fatality rate is reflective of increased testing rates (identifying higher rates of asymptomatic cases). The second drawback is the lack of awareness about environmental conditions of the subject region, they usually provide inaccurate estimates especially during the winter months (13). Several related viral infections, such as the Influenza virus, human coronavirus, and human respiratory, already show notable seasonality (showing peak incidences during only the winter (or summer) months) (14, 15). There are currently several studies that demonstrate the strong dependence of the transmission of SARS-CoV-2 virus on one or more environmental conditions, even after controlling (isolating) the impact of mitigation measures and behavioral changes that reduce contacts (16–21).

To our knowledge, there is currently no model capable of accurately monitoring the current epidemiological situation and predicting future scenarios while considering a reasonably low number of parameters and accounting for the effects of environmental conditions (Table 1).

**Table 1:** Comparison to other models used to inform government policymakers, as of January 2021.

**Model**	**Open source**	**Well documented^**#**^**	**Accounting for seasonality**	**Low number of parameters**	**Reported COVID-19 statistics**
COVIDHunter (this work)	✓	✓	✓	✓	✓ (*R*, cases, hospitalizations, and deaths)
IBZ (11)	✓	**χ**	**χ**	✓	**χ** (only *R*)
LSHTM (7)	✓	**χ**	**χ**	✓	**χ** (only cases)
ICL (9)	✓	✓	**χ**	**χ**	✓ (*R*, cases, hospitalizations, and deaths)
IHME (10)	✓^*^	**χ**	**χ**	**χ**	**χ** (cases, hospitalizations, and deaths)

* *The available packages are configured only for the IHME infrastructure. # Based on the documentation available on each model's GitHub page (all models are available on GitHub)*.

Our **goal** in this work is to develop and validate such a COVID-19 outbreak simulation model. To this end, we introduce COVIDHunter, a simulation model that evaluates the current mitigation measures (i.e., non-pharmaceutical intervention or NPI) that are applied to a region and provides insight into what strength the upcoming mitigation measure should be and for how long it should be applied, while considering the potential effect of environmental conditions. Our model accurately forecasts the numbers of infected and hospitalized patients, and deaths for a given day, as validated on historical COVID-19 data (after accounting for under-reporting). The **key idea** of COVIDHunter is to quantify the spread of COVID-19 in a geographical region by calculating the daily reproduction number, *R*, of COVID-19 and scaling the reproduction number based on changes in mitigation measures, environmental conditions, different variants of concern, and vaccination rate. The *R* number changes during the course of the pandemic due to the change in the ability of a pathogen to establish an infection during a season and mitigation measures that lead to a lower number of susceptible individuals. COVIDHunter simulates the entire population of a region and assigns *each* individual in the population to a stage of the COVID-19 infection (e.g., from being healthy to being short-term immune to COVID-19) based on the scaled *R* number. COVIDHunter requires *only* three input parameters, two of which are time-varying parameters, to calculate the *R* number, which provides four key advantages: (1) allowing flexible (easy-to-adjust) configuration of the model input parameters for different scenarios and different geographical regions; (2) enabling short simulation execution time and simpler modeling; (3) enabling easy validation/correction of the model prediction outcomes by adjusting fewer variables, and (4) being extremely useful and powerful especially during the early stages of a pandemic as many of the parameters are unknown. Whenever applicable, we compare the simulation output of our model to that of four state-of-the-art models currently used to inform policy-makers, IBZ (11), LSHTM (7), ICL (9), and IHME (10).

## Materials and Methods

**Figure 1:**
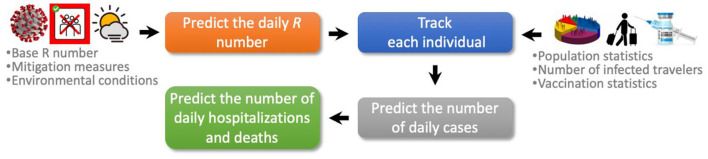
Proposed COVIDHunter model for simulating COVID-19 outbreak.

The COVIDHunter model employs a four-stage approach to simulate the COVID-19 outbreak (Figure 1). (1) Predicting the daily reproduction number, the average number of new daily infections caused by each infected person. (2) COVIDHunter simulates the entire population of a region and labels each individual according to different stages of the COVID-19 infection timeline. Each stage has a different degree of infectiousness and contagiousness. The model simulates these stages for each individual to maintain accurate predictions. (3) Predicting the number of daily cases based on our population simulation. (4) Predicting the number of daily deaths and hospitalizations based on both the predicted number of cases and the *R* number. All input parameters to our model are fully configured based on either existing research findings or user-defined values.

### Predicting the Reproduction Number

One of the most challenging factors in predicting the spread of COVID-19 is to quantify the daily reproduction number (*R*) due to the large number of factors affecting its value and various viral genetic variations. The *R* number is *directly* affected by a large number of factors (12), such as (1) the transmissibility of the virus variant of concern; (2) the strength of the mitigation measures; (3) weather factors (e.g., temperature); (4) air pollutants; (5) population density, and many more. The coronavirus genome can also exhibit rapid genetic changes in its nucleotide sequence (22, 23). This genetic diversity affects the virus virulence, infectivity, transmissibility, and evasion of the host immune responses (23, 24). To provide accurate predictions of the reproduction number, the COVIDHunter model considers only three key factors for predicting the *R* number: (1) different transmissibility rates of infection into a susceptible host population for each SARS-CoV-2 variant, (2) mitigation measures (e.g., lockdown, social distancing, and isolating infected people), and (3) environmental conditions (e.g., air temperature). We choose these three main factors for two reasons: (1) they have a large impact on the *R* number (3, 14, 15), (2) the mitigation measure and the environmental conditions can represent almost any other factor that affects the *R* number (e.g., high population density can be thought of as a weaker mitigation measure). The COVIDHunter model allows for *directly* leveraging existing models that study the effect of *only* mitigation measures (or *only* environmental conditions) on the spread of COVID-19. Our model calculates the time-varying *R* number using **Equation 1** as follows:


(1) $$\begin{array}{r} R\left(t\right)=R0^{*}{\left[1-M\left(t\right)\right]}^{*}C_{e}\left(t\right) \end{array}$$

where *R*0 is the base reproduction number for the virus variant of concern, *M*(*t*) is the mitigation coefficient for the given day *t*, and *C*_*e*_(*t*) is the environmental coefficient for the given day *t*. The *R*0 number quantifies the transmissibility of infection into a susceptible host population by calculating the expected average number of new infections caused by an infected person in a population with no prior immunity to a specific virus or variant (as a pandemic virus is by definition novel to all populations). Hence, the *R*0 number represents the transmissibility of an infection at only the beginning of the outbreak assuming the population is not protected via vaccination. Unlike the *R* number, the *R*0 number is a fixed value and it does not depend on time. The *R*0 number for each SARS-CoV-2 variant can be obtained from several existing studies [such as in (25–28)] that estimate it by modeling contact patterns during the first wave of the pandemic.

The mitigation coefficient [*M*(*t*)] applied to the population is a time-dependent variable and it has a value between 0 and 1, where 1 represents the strongest mitigation measure and 0 represents no mitigation measure applied. In different countries, mitigation measures take different forms, such as social distancing, self-isolation, school closure, banning public events, and complete lockdown. These measures exhibit significant heterogeneity and differ in timing and intensity across countries (9). *The Oxford Stringency Index* (29) maintains a twice-weekly-updated index that represents the severity of nine mitigation measures that are applied by more than 160 countries. Another study (30) estimates the effect of *only* seven mitigation measures on the *R* number in 41 countries. We can *directly* leverage such studies for calculating the mitigation coefficient on a given day.

The environmental coefficient [*C*_*e*_(*t*)] is a time-dependent variable representing the effect of external environmental factors on the spread of COVID-19 and it has a value between 0 and 2. Several studies have demonstrated increased infectiousness by a country-dependent fixed-rate with each 1 °C fall in daytime temperature (16, 17). Another study supports the same temperature-infectiousness relationship, but it also finds that before applying any mitigation measures, a one-degree drop in relative humidity shows increased infectiousness by a rate lower (2.94× less) than that of temperature (19). Another study follows a simple way of modeling the effect of seasonality on COVID-19 transmission using a sinusoidal function with an annual period (20). One of the most comprehensive studies that spans more than 3,700 locations around the world is *HARVARD CRW* (or CRW in short) (21). It finds the statistical correlation between the relative changes in the *R* number and both weather conditions and air pollution after controlling the impact of mitigation measures. Our model enables applying *any of these studies* as we experimentally demonstrate in **Section Result**. In our experiments, we choose two main approaches for setting the value of the time-varying environmental coefficient variable [*C*_*e*_(*t*)]. (1) The first approach is to perform statistical analysis for the relationship between the daily number of COVID-19 cases and average daytime temperature in Switzerland. (2) The second approach is to apply the *HARVARD CRW* (21) (referred to as *CRW*). Next, we explain the first approach in detail.

### Statistical Relationship Between Temperature and Number of COVID-19 Cases

To calculate the environmental coefficient, we explore the relationship between the daily new confirmed COVID-19 case counts or death counts and temperature in Switzerland. We obtain the daily number of confirmed COVID-19 cases and deaths in Switzerland from official reports of the Federal Office of Public Health (FOPH) in Switzerland (31) starting from March 2020 until January 2021. We obtain the air temperature data from the Federal Office of Meteorology and Climatology (MeteoSwiss) in Switzerland (32). We calculate the daily average air temperature during the same time period (March 2020 to December 2020) for all the 26 cantons in Switzerland. To evaluate the correlation between the temperature data and the number of daily confirmed COVID-19 cases or the daily counts of death, we use a generalized additive model (GAM). GAM is usually used to calculate the linear and non-linear regression models between meteorological factors (e.g., temperature, humidity) with COVID-19 infection and transmission (16, 17, 33).

**Figure 2:**
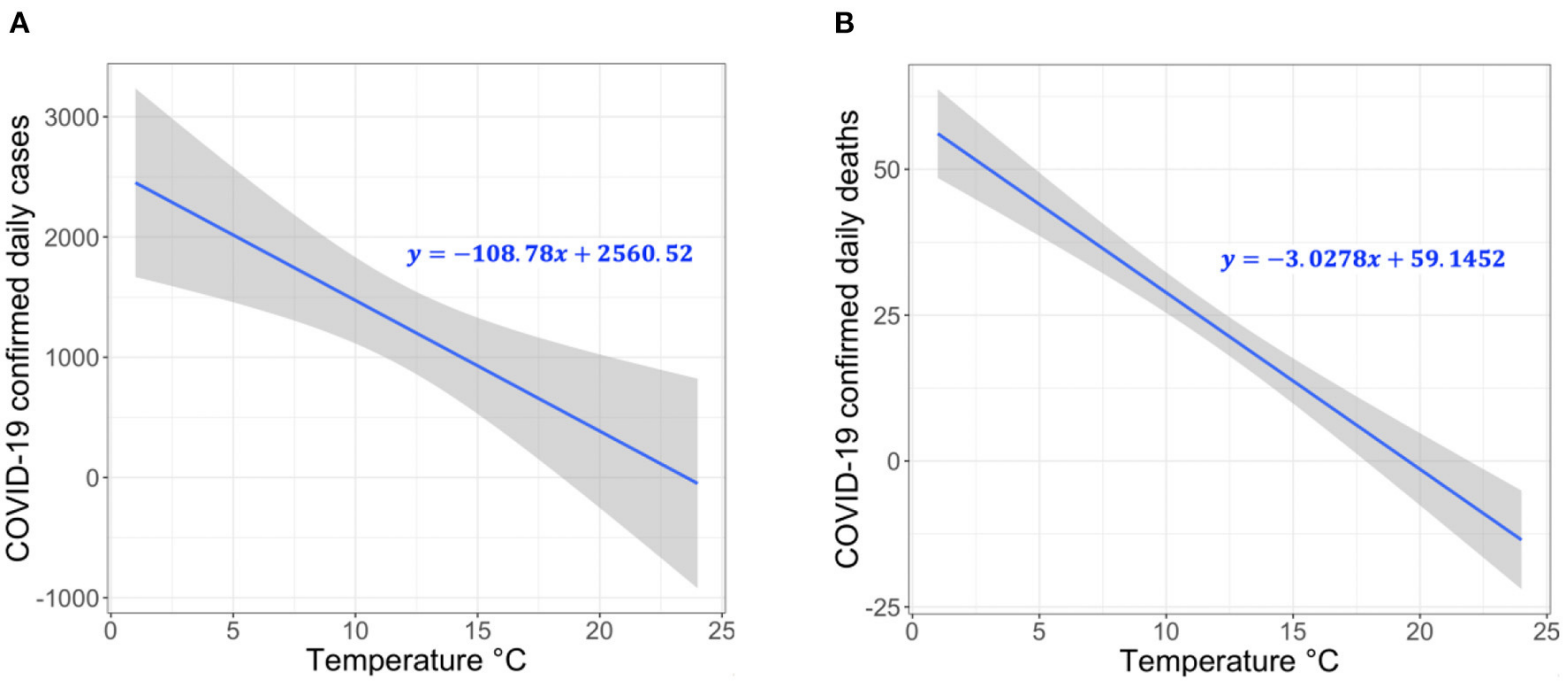
Correlation between temperature and COVID-19 confirmed **(A)** case count and **(B)** death count in 26 cantons of Switzerland.

Our analyses are performed with R software version 4.0.3, where *p*–value <0.05 is considered statistically significant. Our model attempts to represent the linear behavior of the growth curve of the counts of the new confirmed cases or deaths in Switzerland. Therefore, we can test the hypothesis of whether there is a significant negative correlation between the COVID-19 confirmed daily case or death counts and temperature. The results demonstrate a significant negative correlation between temperature and COVID-19 daily case and death counts. Specifically, the relationship is linear for the average temperature in the range from 1–26°C. Based on Figure 2, we make two key observations. (1) For each 1°C rise in temperature, there is a 3.67% (*t*-value = 3.244 and *p*-value = 0.0013) decrease in the daily number of COVID-19 confirmed cases (Figure 2A). (2) For each 1°C rise in temperature, there is a 23.8% decrease in the daily number of COVID-19 deaths (*t*-value = 9.312 and *p*-value = 0.0), as shown in Figure 2B. The statistical analysis can be reproduced using the following script https://github.com/CMU-SAFARI/COVIDHunter/tree/main/TemperatureSensitivityStudy.

### Labeling Each Individual in the Subject Population According to Different Stages of the COVID-19 Infection Timeline

**Figure 3:**
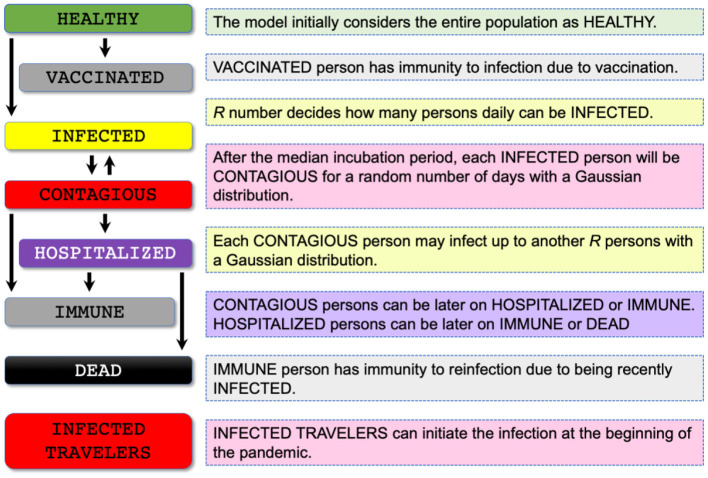
Proposed population clustering algorithm for assigning each individual in the population of concern to a stage of the COVID-19 infection timeline. The COVIDHunter model makes eight main clusters: HEALTHY, VACCINATED, INFECTED, CONTAGIOUS, HOSPITALIZED, IMMUNE, DEAD, and INFECTED TRAVELERS.

COVIDHunter tracks the number of infected and uninfected persons over time by clustering the population into eight main categories: HEALTHY, VACCINATED, INFECTED, CONTAGIOUS, HOSPITALIZED, IMMUNE, DEAD, and INFECTED TRAVELERS (Figure 3). The model initially considers the entire population as uninfected (i.e., HEALTHY). For each simulated day, the COVIDHunter model decides which persons will have immunity to infection due to vaccination (i.e., VACCINATED) based on input data. For the unvaccinated persons, the model calculates the *R* value using **Equation 1** (**Section Predicting the reproduction number**) and decides how many persons can be infected (i.e., INFECTED) during each simulated day. Our modeling approach considers multiple virus strains/variants by calculating multiple *R* numbers, each of which represents a different virus strain/variant. The day when the first case of infection (caused by a variant of concern) in a population introduced is defined by the user. For each newly infected person (INFECTED), the model maintains a counter that counts the number of days from being infected to being contagious (CONTAGIOUS). Several COVID-19 case studies show that *presymptomatic* transmission can occur 1–3 days before symptom onset (34, 35). COVID-19 patients can develop symptoms mostly after an incubation period of 1 to 14 days (the median incubation period is estimated to be 4.5 to 5.8 days) (4, 5). We calculate the number of days of being contagious after being infected as a random number with a Gaussian distribution that has user-defined lowest and highest values. Each contagious person may infect *N* other persons depending on mobility, population density, number of households, and several other factors (36). We calculate the value of *N* to be a random number with a Gaussian distribution that has the lowest value of 0 and the highest value determined by the user. If *N* is greater than the *R* number (i.e., the target number of infections for that day has been reached), further infections are curtailed preventing overestimation of *N* by infecting *only* *R* persons.

Once the contagious person infects the desired number of susceptible persons, the status of the contagious person becomes IMMUNE or HOSPITALIZED. The IMMUNE status indicates that the person has immunity to reinfection due to either vaccination or being recently infected (37, 38). The HOSPITALIZED person can be later on IMMUNE or DEAD. There are currently two key approaches for calculating the estimated number of both hospitalizations and deaths due to COVID-19: (1) using historical statistical probabilities, each of which is unique to each age group in a population (39, 40) and (2) using historical COVID-19 hospitalizations-to-cases and deaths-to-cases ratios (41). We choose to follow the second approach as it does *not* require (1) clustering the population into age-groups and (2) calculating the risk of each individual using the given probability, which both affect the complexity of the model and the simulation time. As the *true* number of cases is unknown due to both lack of population-scale testing and asymptomatic cases (42, 43), it is extremely difficult to make accurate estimates of the *true* number of COVID-19 hospitalizations and deaths. As such, we assume a fixed multiplicative relationship between the number of laboratory-confirmed cases and the *true* number of cases. We use user-defined correction coefficients (we refer to them as *certainty rate levels*) to account for such a multiplicative relationship. A certainty rate of, for example, 50% means that the *true* number of COVID-19 cases is actually *double* that calculated by COVIDHunter.

Our model also simulates the effect of infected travelers (i.e., INFECTED TRAVELERS) on the value of *R*. These travelers (e.g., daily cross-border commuters within the European Union) can initiate the infection(s) at the beginning of the pandemic. If such infected travelers are absent (due to, for example, emergency lockdown) from the target population, the virus would die out once the value of *R* decreases below one for a sufficient period of time. The percentage of incoming infected travelers is *not* affected by the changes in the local mitigation measures nor the environmental conditions, as these travelers were already infected abroad.

### Predicting the Number of COVID-19 Cases

The COVIDHunter model assigns each individual in the entire population of a region to a stage of the COVID-19 infection timeline. Using this assignment, our model predicts the *daily* number of COVID-19 cases for a given day *t*, as follows:


(2) $$\begin{array}{r} Daily\text{\_}Cases\left(t\right)=\overset{T_{INF}\left(t\right)}{\underset{n=0}{\sum }}N\left(n\right)+\overset{U_{CON}\left(t\right)}{\underset{m=0}{\sum }}N\left(m\right) \end{array}$$

where *T*_*INF*_ is the daily number of infected travelers that is a user-defined variable, *N*() is a function that calculates the number of persons to be infected by a given person as a random number with a Gaussian distribution, and *U*_*CON*_ is the daily number of contagious persons calculated by our model.

### Predicting the Number of COVID-19 Hospitalizations and Deaths

The number of COVID-19 hospitalizations for a given day, *t*, can be calculated as follows:


(3) $$\begin{array}{r} Daily\text{\_}Hospitalizations\left(t\right)=Daily\text{\_}Cases{\left(t\right)}^{*}X^{*}C_{X} \end{array}$$

where *Daily*_*Cases*(*t*) is calculated using **Equation 2** and *X* is the hospitalizations-to-cases ratio that is calculated as the average of daily ratios of the number of COVID-19 hospitalizations to the laboratory-confirmed number of COVID-19 cases. As the *true* number of cases is unknown due to both lack of population-scale testing and asymptomatic cases (42, 43), it is extremely difficult to make accurate estimates of the *true* number of COVID-19 hospitalizations. As such, we assume a fixed multiplicative relationship between the number of laboratory-confirmed cases and the *true* number of cases. We use the user-defined correction coefficient, *C*_*X*_, of the hospitalizations-to-cases ratio to account for such a multiplicative relationship. The number of COVID-19 deaths for a given day *t* can be calculated as follows:


(4) $$\begin{array}{r} Daily\text{\_}Deaths\left(t\right)=Daily\text{\_}Cases{\left(t\right)}^{*}Y^{*}C_{Y} \end{array}$$

where *Daily*_*Cases*(*t*) is calculated using **Equation 2** and *Y* is the deaths-to-cases ratio, which is calculated as the average of daily ratios of the number of COVID-19 deaths to the number of COVID-19 laboratory-confirmed cases. The observed number of COVID-19 deaths can still be less than the *true* number of COVID-19 deaths due to, for example, under-reporting. We use the user-defined correction coefficient, *C*_*Y*_, to account for the under-reporting. One way to find the *true* number of COVID-19 deaths is to calculate the number of excess deaths. The number of excess deaths is the difference between the observed number of deaths during a time period and the expected (based on historical data) number of deaths during the same time period. For this reason, *C*_*Y*_ may not necessarily be equal to *C*_*X*_.

## Results

We evaluate the daily (1) *R* number, (2) mitigation measures, and (3) numbers of COVID-19 cases, hospitalizations, and deaths. We compare the predicted values to their corresponding observed values and that of four state-of-the-art models, ICL (9), IHME (10), IBZ (11), and LSHTM (7), whenever possible. We provide a comprehensive treatment of *all* datasets, models, and evaluation results with different model configurations in the Supplementary Materials and on GitHub page of COVIDHunter, https://github.com/CMU-SAFARI/COVIDHunter. We also provide all parameter values used for running COVIDHunter and different scripts for reproducing the experimental evaluation performed in this work on our GitHub page, https://github.com/CMU-SAFARI/COVIDHunter/tree/main/Reproduce-Switzerland-Case-Study-Results. We provide below our prediction run for the period of 20 November 2021 until February 2022, which was carried out on 20 November 2021. We provide another prediction run for the period from 19 April 2021 until 1 June 2021 in the Supplementary Materials, which were carried out on 19 April 2021. We also provide a comprehensive analysis of the COVID-19 statistics provided by ICL (9), IHME (10), IBZ (11), and LSHTM (7) from the beginning of the COVID-19 outbreak (February 2020) until April 2021.

### Determining the Value of Each Variable in the Equations

We use Switzerland as a use-case for all the experiments. However, our model is not limited to any specific region as the parameters it uses are completely configurable. To predict the *R* number, we use **Equation 1** that requires three key variables. We set the base reproduction numbers, *R*0, for two main variants, the Delta variant and its ancestral strain, of SARS-CoV-2 in Switzerland as 5 and 2.7, respectively, as shown in (25, 26, 44). The recent Omicron variant was not circulating during the study. We set the first day for the Delta variant to be injected into the population as 19 June 2021 based on the governmental data (45). We set the first day of vaccination availability in Switzerland as 28 February 2021, with a vaccination rate of 0.28 per day based on governmental data (45). We change the daily mitigation coefficient, *M*(*t*), value based on the ratio of number of confirmed hospitalizations to the number of confirmed cases with two certainty rate levels of 100 and 50%, as we explain in detail in **Section Model validation**. This helps us to take into account uncertainty in the observed number of COVID-19 cases, hospitalizations, and deaths. We set the minimum and maximum incubation time for SARS-CoV-2 as 1 and 5 days, respectively, as 5-day period represents the median incubation period worldwide (4, 5). We set the population of Switzerland to 8,654,622. We empirically choose the values of *N*, the number of travelers, and the ratio of the number of infected travelers to the total number of travelers to be 25, 100, and 15%, respectively.

### Model Validation

We can validate our model using two key approaches. (1) Comparing the daily *R* number predicted by our model (using **Equation 1**) with the daily reported official *R* number for the same region. (2) Comparing the daily number of COVID-19 cases predicted by our model (using **Equation 2**) with the daily number of laboratory-confirmed COVID-19 cases. We decide to use a combination of reported numbers of cases, hospitalizations, and deaths to validate our model for three main reasons. (1) The *R* number is calculated as, for example, the ratio of the number of cases for a week (7-day rolling average) to the number of cases for the preceding week. Adjusting the parameters of our model to fit the curve of the number of confirmed cases is likely to be highly uncertain. (2) The reported daily reproduction number by authorities of Switzerland usually excludes the values for the last 14 days, which makes the validation based on the reproduction number more challenging. (3) As of 2022, we have already witnessed more than two years of the pandemic, which provide us with several observations and lessons. The most obvious source of uncertainty, affecting *all* models, is that the *true* number of persons that are previously infected or currently infected is *unknown* (46). However, the publicly-available number of COVID-19 hospitalizations and deaths can provide more reliable data.

We validate our model using three key steps. (1) We leverage the more reliable data of reported number of hospitalizations (or deaths) to estimate the *true* number of COVID-19 cases using the ratio of number of laboratory-confirmed hospitalizations (or deaths) to the number of laboratory-confirmed cases during the second wave of the COVID-19 pandemic. We assume that the COVID-19 statistics during the second wave is more accurate than that during the first wave because generally more testing is performed in the second wave. (2) We consider a multiplicative relationship between the *true* number of COVID-19 cases and that estimated in step 1. In our experimental evaluation, we use the *true* number of COVID-19 cases calculated using different multiplicative factor values (we refer to them as *certainty rate levels*) as a ground-truth for validating our model. A certainty rate of, for example, 50% means that the *true* number of COVID-19 cases is actually *double* that calculated in step 1. (3) We use our model to calculate both the daily *R* number (**Equation 1**) and the number of COVID-19 cases (**Equation 2**). We fix the two terms of **Equation 1**, *R*0 and *C*_*e*_, using publicly-available data for a given region and change the third term, *M*, until we fit the curve of the number of cases predicted by our model to the ground-truth plot calculated in step 2.

### Evaluating the Expected Number of COVID-19 Cases for Model Validation

**Figure 4:**
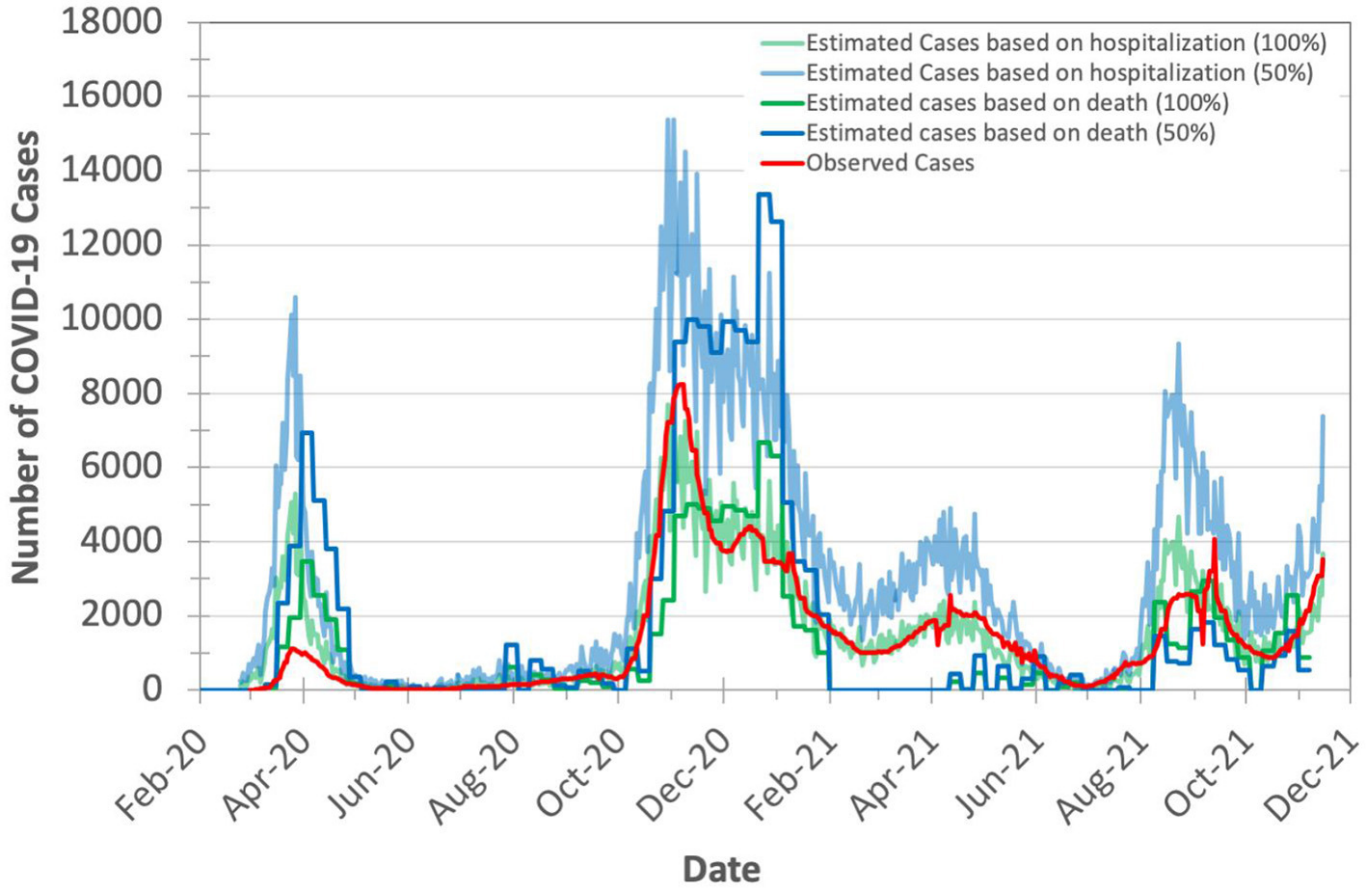
Observed (officially reported) and expected number of COVID-19 cases in Switzerland during the years 2020 and 2021. We calculate the expected number of cases based on both the hospitalizations-to-cases and deaths-to-cases ratios for the second wave. We assume two certainty rate levels of 50 and 100%.

As the exact true number of COVID-19 cases remains unknown (due to, for example, lack of population-scale COVID-19 testing), we expect the true number of COVID-19 cases in Switzerland to be higher than the observed (laboratory-confirmed) number of cases. We calculate the expected true number of cases based on both numbers of deaths and hospitalizations, as we explain in **Section Model validation**. To account for the possible missing number of COVID-19 deaths, we consider the excess deaths instead of observed deaths. We calculate the excess deaths as the difference between the 5-year average of weekly deaths and the observed weekly number of deaths in both 2020 and 2021. We find that *X* (hospitalizations-to-cases ratio) and *Y* (deaths-to-cases ratio, using excess death data) to be 3.75 and 2.441%, respectively, during the second wave of the pandemic in Switzerland. We choose the second wave to calculate the values of *X* and *Y* as Switzerland has increased the daily number of COVID-19 testing by 5.31× (21,641/4,074) on average compared to the first wave. We calculate the expected number of cases on a given day *t* with certainty rate levels of 100 and 50% based on hospitalizations by dividing the number of hospitalizations at *t* by *X* and *X*/2, respectively, as we show in Figure 4. We apply the same approach to calculate the expected number of cases on a given day *t* with certainty rate levels of 100% and 50% based on deaths using *Y* and *Y*/2, respectively.

Based on Figure 4, we make three key observations. (1) The plot for the expected number of cases calculated based on the number of deaths is shifted forward by 10–20 days (15 days on average) from that for the expected number of cases calculated based on the number of hospitalizations. This is due to the fact that each hospitalized patient usually spends some number of days in the hospital before dying of COVID-19. We do not observe a significant time shift between the plot of the expected number of cases calculated based on the number of hospitalizations and the plot of observed (laboratory-confirmed) cases. (2) The expected number of cases calculated based on the number of excess deaths is not reliable when the mass COVID-19 vaccination is kicked-off (after February 2021) as the number of deaths is quickly declined. (3) The expected number of cases calculated based on the number of hospitalizations is on average 2.7× higher than the expected number of cases calculated based on the number of excess deaths (after accounting for the 15-day shift) for the same certainty rate. This is expected as not all hospitalized patients die.

We conclude that the number of COVID-19 hospitalizations can be used reliably for estimating the true number of COVID-19 cases.

### Evaluating the Predicted Number of COVID-19 Cases

**Figure 5:**
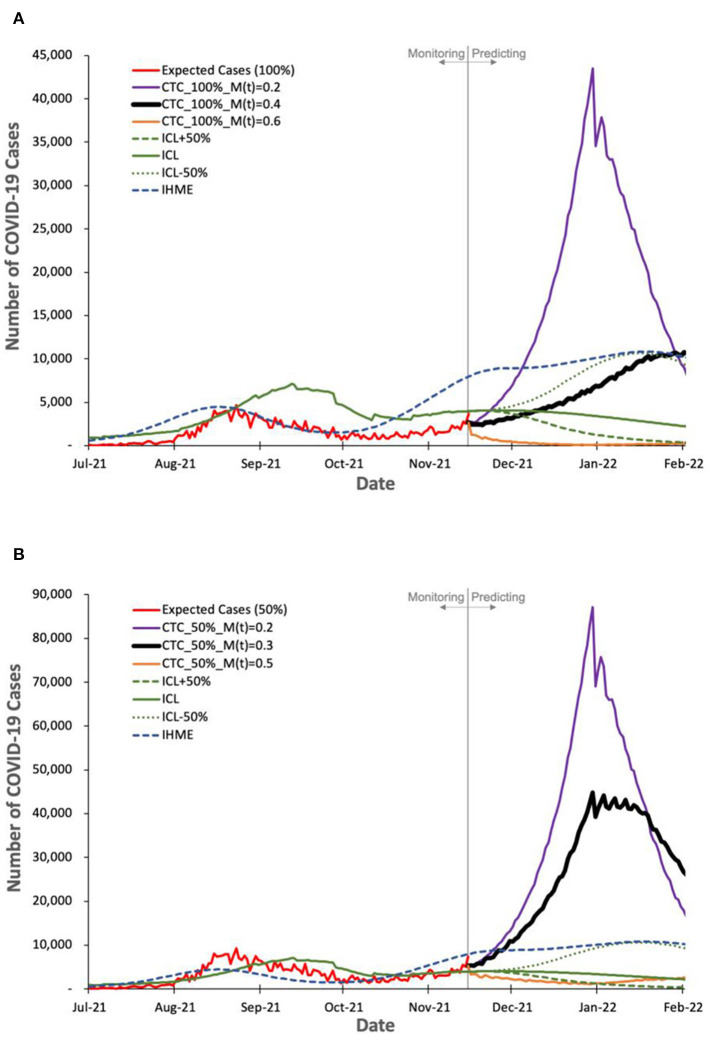
Observed and predicted number of COVID-19 cases by our model and other two state-of-the-art models, ICL and IHME. For COVIDHunter, we use CTC environmental condition approaches with two certainty rate levels of **(A)** 100% and **(B)** 50%. We show the prediction of COVIDHunter using three mitigation coefficient, *M*(*t*), values, each of which is applied from 20 November to 20 December 2021. The predicted plot in a bold black line represents the situation when the mitigation measures applied before the prediction period remain the same.

We evaluate COVIDHunter's *predicted* daily number of COVID-19 cases in Switzerland. We compare the predicted numbers by our model to the observed numbers and those provided by two state-of-the-art models (ICL and IHME), as shown in Figures 5A,B. We calculate the observed number of cases as the expected number of cases with a certainty rate level of 100% (as we discuss in **Section Evaluating the expected number of COVID-19 cases for model validation**). We use three default configurations for the prediction of the ICL model: (1) strengthening mitigation measures by 50%, (2) maintaining the same mitigation measures, and (3) relaxing mitigation measures by 50% which we refer to as ICL+50%, ICL, and ICL-50%, respectively, in Figure 5. We use the mean numbers reported by the IHME model. As we provide in **Section Statistical relationship between temperature and number of COVID-19 cases**, our statistical analysis shows that each 1°C rise in daytime temperature is associated with a 3.67% (*t*-value = −3.244 and *p*-value = 0.0013) decrease in the daily number of confirmed COVID-19 cases. We refer to this approach as Cases-Temperature Coefficient (CTC).

Based on Figures 5A,B we make three key observations. (1) Our model predicts that the peak (the highest number of COVID-19 cases) of the upcoming wave will be on 26 January 2022 (reaching up to 10,000 daily cases) and 31 December 2021 (reaching up to 44,800 daily cases and peaking up to 17 January 2022) for a certainty rate levels of 100% (Figure 5A) and 50% (Figure 5B), respectively, while maintaining the same strength of the current (20 November 2021) mitigation measures for 30 days. Both IHME and ICL models consider that the current number of COVID-19 cases in Switzerland shows a certainty rate level of 50% and the highest number of daily cases will be 10,000, but IHME and ICL models predict the peak of the upcoming wave to be on 26 January 2022 and 16 December 2021, respectively. (2) The number of COVID-19 cases reduces from 10,000 to 200 daily cases and from 44,800 to 2,400 daily cases for a certainty rate levels of 100% (Figure 5A) and 50% (Figure 5B), respectively, within January 2021 if the mitigation measures that are applied nationwide in Switzerland are tightened by 50% [*M(t)* increases from 0.4 to 0.6 and from 0.3 to 0.5, respectively] for at least 30 days starting from 20 November to 20 December 2021. (3) Relaxing the mitigation measures before at least February 2022 can lead to a significant rise in the number of daily COVID-19 cases, reaching up to 43,500 as predicted by ICL and COVIDHunter (certainty rate levels of 100%) and up to 82,900 daily cases as predicted by COVIDHunter (certainty rate levels of 50%).

### Evaluating the Predicted Number of COVID-19 Hospitalizations and Deaths

**Figure 6:**
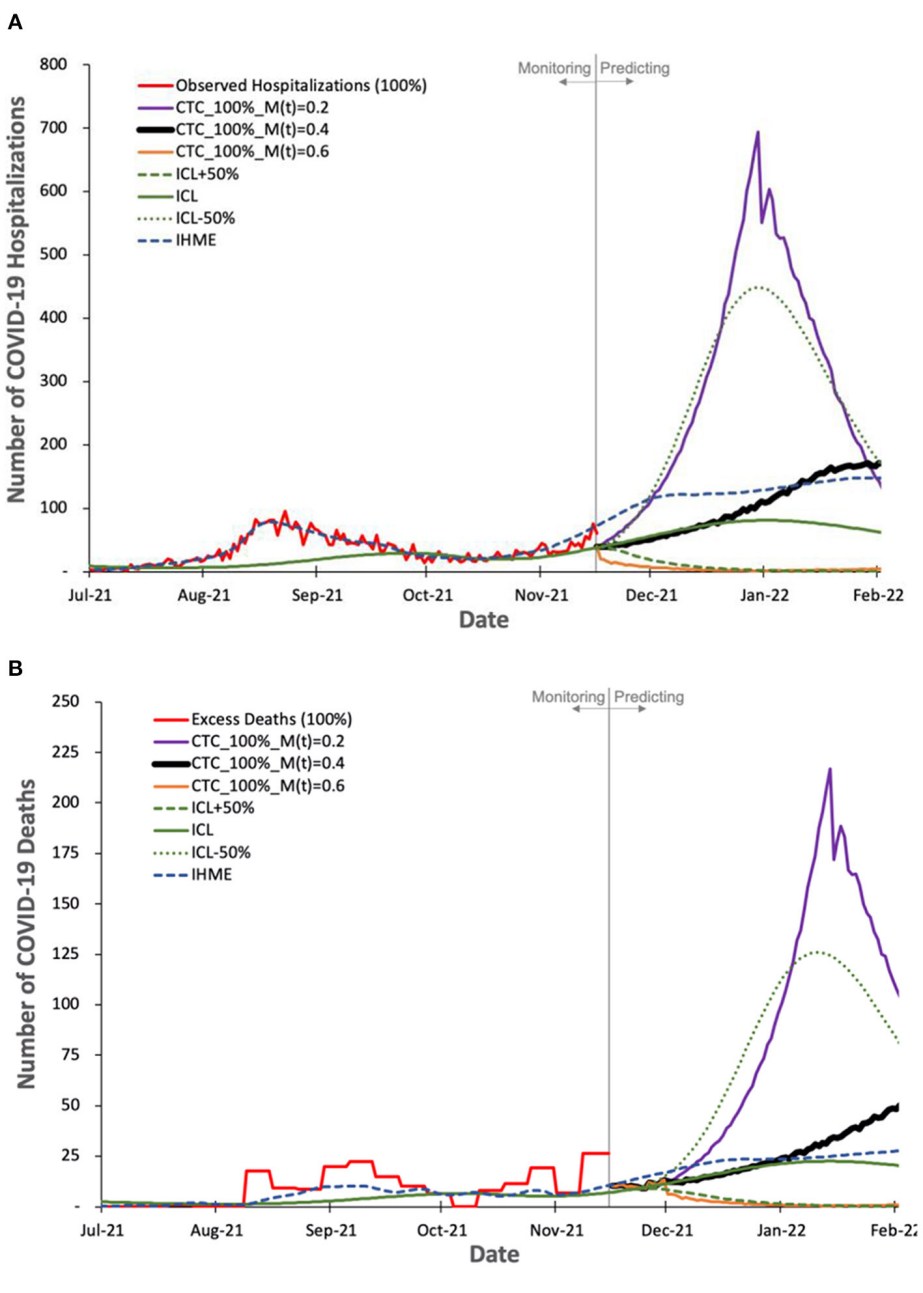
Observed and predicted number of COVID-19 hospitalization and deaths by our model and other two state-of-the-art models, ICL and IHME. For COVIDHunter, we use a certainty rate level of 100% for the numbers of **(A)** hospitalizations and **(B)** deaths, as IHME and ICL models tend to follow such a certainty rate. We show the prediction of COVIDHunter using three mitigation coefficient, *M*(*t*), values, each of which is applied from 20 November to 20 December 2021. The predicted plot in a bold black line represents the situation when the mitigation measures applied before the prediction period remain the same.

We evaluate COVIDHunter's *predicted* daily number of COVID-19 hospitalizations and deaths in Figures 6A,B. We use the observed official number of hospitalizations as is. We calculate the observed number of deaths as the number of excess deaths to account for uncertainty in reporting COVID-19 deaths. Using the number of cases calculated with **Equation 2** and the observed number of hospitalizations and excess deaths (after accounting for 15-day shift, as we discuss in **Section Evaluating the predicted number of COVID-19 cases** and Figure 4) during 1 August 2021 to 15 November 2021, we find *X* (hospitalizations-to-cases ratio) and *Y* (deaths-to-cases ratio, using excess death data) to be 1.508 and 0.498%, respectively. We choose the period from 1 August 2021 to 15 November 2021 for calculating the *X* and *Y*ratios to provide accurate predictions since the vaccination rate in Switzerland exceeds 50%, most of the risk groups received their second vaccination dose, and the Delta variant dominates the causes for COVID-19 cases (47).

Based on Figures 6A,B we make four key observations. (1) IHME and ICL models consider that the current numbers of COVID-19 hospitalizations and deaths in Switzerland show a certainty rate level of 100%. (2) COVIDHunter and IHME show that the highest number of hospitalizations and deaths will be on 26 January 2022 (reaching up to 160 and 44 daily hospitalizations and deaths, respectively), which is a month and 2 weeks after that predicted by ICL for the number of hospitalizations and deaths, respectively. These predictions show that we will face a similar situation to the first wave we had in February 2020 if we maintain the same current mitigation measures. (3) COVIDHunter and ICL models show that relaxing the current mitigation measures by 50% for a month (20 November to 20 December 2021) can increase the numbers of hospitalizations and deaths by up to 5.5x. They also show that tightening the current mitigation measures by 50% for a month (20 November to 20 December 2021) can reduce the numbers of hospitalizations and deaths by up to 3.9x.

### Evaluating the Prediction Accuracy

**Figure 7:**
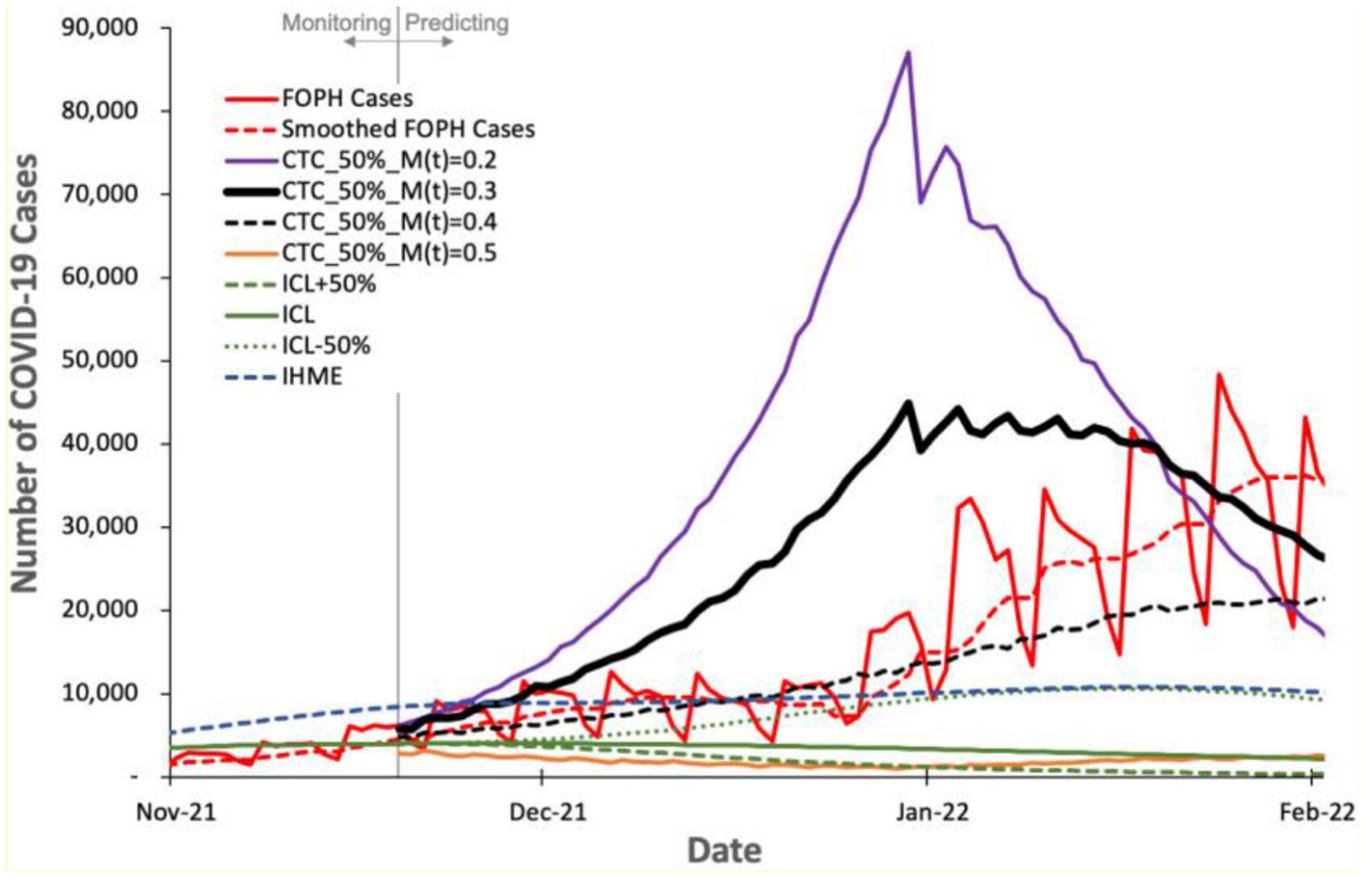
Predicted number of COVID-19 cases by COVIDHunter model and other two state-of-the-art models, ICL and IHME, compared to the real number (FOPH Cases) of COVID-19 cases released after performing the prediction.

**Figure 8:**
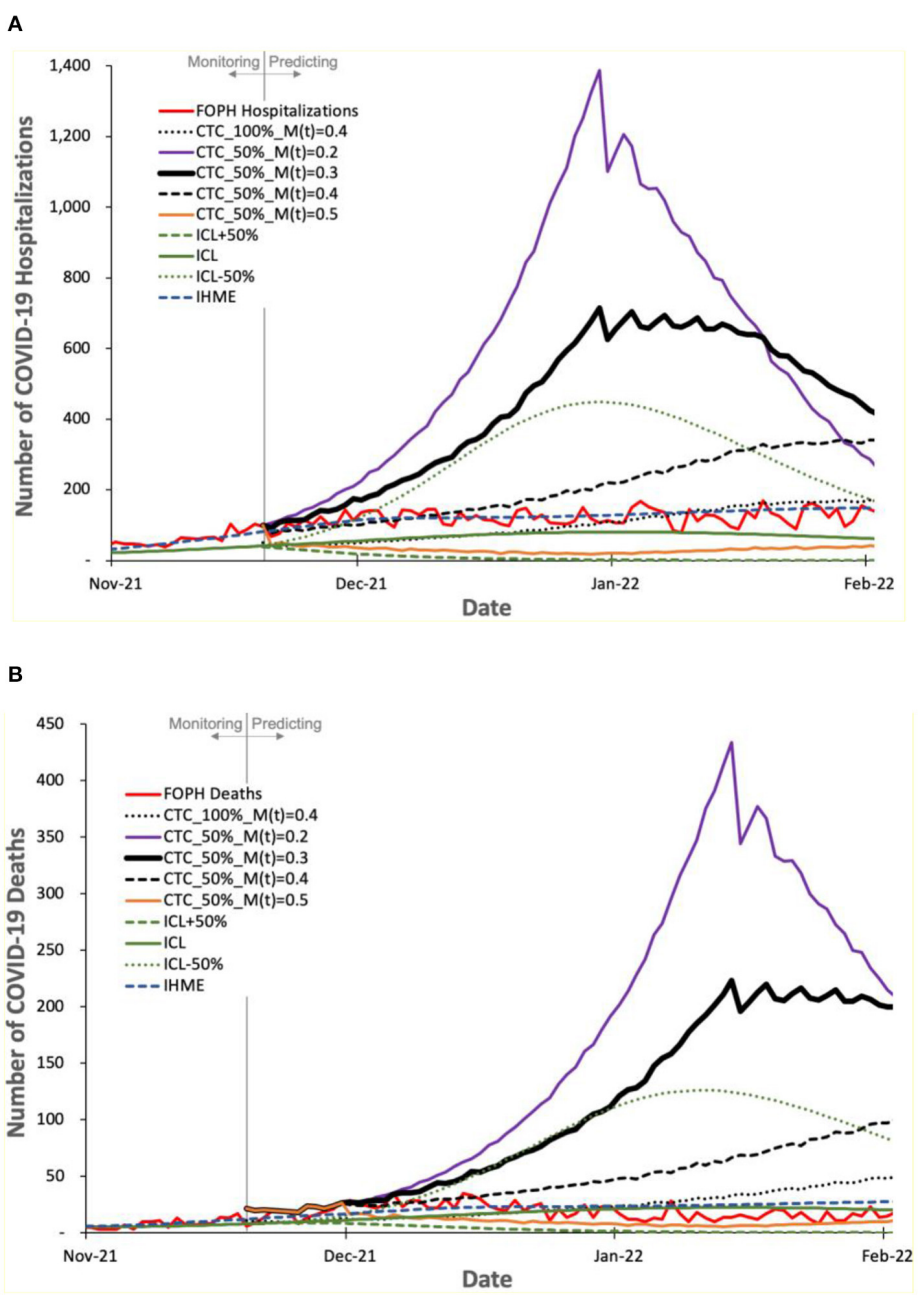
Predicted number of COVID-19 **(A)** hospitalizations and **(B)** deaths by COVIDHunter, ICL, and IHME, compared to the real numbers (FOPH Hospitalizations and FOPH Deaths) released after performing the prediction.

We evaluate the prediction accuracy of COVIDHunter, ICL, and IHME models using the real COVID-19 statistics that are published by the Federal Office of Public Health (FOPH) of Switzerland three months after performing the prediction. We evaluate the prediction accuracy for the number of cases, hospitalizations, and deaths due to COVID-19 in Figures 7 and 8.

Figure 7 shows the number of COVID-19 cases predicted by the three models and the real number (called “FOPH Cases” in Figure 7) of COVID-19 cases released by FOPH. FOPH usually does not report COVID-19 statistics during the weekends and thus we also show the 7-day rolling average numbers (called “Smoothed FOPH Cases” in Figure 7) of COVID-19 cases as provided by https://ourworldindata.org (referred to as “Smoothed data” in the Supplementary Materials, Section S1). We make four key observations. (1) COVIDHunter is the only model that is able to accurately predict the number of COVID-19 cases. Although COVIDHuner predicts the mitigation measures applied during November 2021 to be of strength 0.3 using a certainty rate level of 50% (Figure 5B), the mitigation measures have been already tightened during November and December 2021, as shown in (48, 49). This causes the real number of COVID-19 cases to match the COVIDHunter's predicted number of cases using a mitigation measure strength of 0.4. This informs us that the mitigation measures are further strengthened from 0.3 to 0.4, which is in line with the actual mitigation measures taken in Switzerland. (2) Even with the increase in the strength of the mitigation measures during November and December 2021, the number of COVID-19 cases keeps increasing after January 2022. We believe this is mainly because of the new variant, Omicron, that starts circulating in the population of Switzerland around the start of December 2021 (50). (3) The IHME's predicted number of cases also matches that of the FOPH's number of cases. However, this indicates that the IHME model provides an inaccurate prediction (i.e., underestimation) as IHME provides the predicted number of COVID-19 cases assuming the strength of the mitigation measures during November and December 2021 to remain the same as that applied before November 2021, which is incorrect based on governmental information (48, 49). (4) The ICL model provides a significantly underestimated number of cases even when the ICL model is configured for increased strength of the mitigation measures by 50% (ICL+50%).

Figure 8 shows the number of COVID-19 hospitalizations and deaths predicted by the three models and the real numbers released by FOPH, called “FOPH Hospitalizations” and “FOPH Deaths”, respectively. We make four key observations. (1) The FOPH's numbers of COVID-19 hospitalizations and deaths show a certainty rate level of 50%. (2) The COVIDHunter model with a certainty rate level of 50% and an increase in the mitigation measure strength from 0.3 to 0.4 provides an accurate prediction of both the number of hospitalizations and the number of deaths, which is in line with the real numbers provided by FOPH until a new variant, Omicron, is introduced. (3) The Omicron variant, subsequent increases in mitigation measure strength, and increase in vaccination rate cause fewer hospitalizations and deaths than that predicted by COVIDHunter after January 2022. We did not configure COVIDHunter to account for the Omicron variant when we perform the prediction since the Omicron variant was not a variant of concern in November 2021. (4) Similar to the third and fourth observations we make for Figure 7, we observe that both ICL and IHME provide inaccurate predictions. That is the ICL model still provides significantly underestimated statistics and IHME provides predictions that match the FOPH's numbers, which we believe is implausible as the circumstances of virus variant, mitigation measures, and vaccination rates during January 2022 are very different from that in November 2021.

We conclude that choosing the appropriate configurations for COVIDHunter leads to accurate predictions of numbers of cases, hospitalizations, and deaths. We demonstrate that COVIDHunter is more accurate than state-of-the-art prediction models, ICL and IHME.

## Conclusion and Discussion

We conclude that COVIDHunter provides a more accurate estimation of the number of COVID-19 cases, compared to IHME (which provides inaccurate estimation during the first wave) and ICL (which provides over-estimation), with complete control over the certainty rate level, mitigation measures, and environmental conditions. Unlike LSHTM, COVIDHunter also ensures no prediction delay. We demonstrate the effectiveness of COVIDHunter through about 2 years of monitoring COVID-19 and two prediction runs as we provide in **Section Result** and the Supplementary Materials (Supplemental Figures S1–S5). COVIDHunter gains these unique advantages over existing models by considering environmental conditions, transmissibility of different variants, and vaccination statistics in our modeling.

Using COVIDHunter, we demonstrate that curbing the spread of COVID-19 in Switzerland requires applying stricter mitigation measures than that of the currently applied mitigation measures for at least 30 days. If the authorities maintain the current mitigation measures, we will face another wave that is very similar to the first wave we had in February 2020. Relaxing the mitigation measures should not be an option before at least February 2022. We provide insights on the effect of each change in the strength of the applied mitigation measure on the number of daily cases, hospitalizations, and deaths. We make all the data, statistical analyses, and a well-documented model implementation publicly and freely available to enable full reproducibility and help society and decision-makers.

We especially build COVIDHunter model to be flexible to configure and easy to extend for representing any existing or future scenario using different values of the three terms of **Equation 1**, 1) *R*0, 2) *M*(*t*), 3) *C*_*e*_(*t*), in addition to several other parameters such as different variants of concern, vaccination rate, population, number of travelers, percentage of expected infected travelers to the total number of travelers, and hospitalizations- or deaths-to-cases ratios. The COVIDHunter model considers each location independently of other locations, but it also accounts for potential movement between locations by adjusting the corresponding parameters for travelers. By allowing most of the parameters to vary in time, *t*, the COVIDHunter model is capable of accounting for any change in transmission intensity due to changes in environmental conditions and mitigation measures over time. The flexibility of configuring the environmental coefficient and mitigation coefficient allows our proposed model to control for location-specific differences in population density, cultural practices, age distribution, and time-variant mitigation responses in each location.

COVIDHunter has three main limitations that can be addressed in future work. (1) Our modeling approach acts across the overall population without assuming any specific age structure for transmission dynamics. It is still *possible* to consider each age group separately using individual runs of COVIDHunter model simulation, each of which has its own parameter values adjusted for the target age group. (2) The current implementation of COVIDHunter considers only two variants of concerns at the time. (3) COVIDHunter does not consider different types of vaccines nor different immunity/protection periods after vaccination. Instead COVIDHunter treats all types of vaccines equally and it considers only the vaccination rate per day.

## Data Availability Statement

The datasets presented in this study can be found in online repositories. The names of the repository/repositories and accession number(s) can be found below: https://github.com/CMU-SAFARI/COVIDHunter/tree/main/Reproduce-Switzerland-Case-Study-Results.

## Author Contributions

MA, ST, and OM led the project. NA performed the statistical analysis. MA and JK produced the tables and figures. MA, JK, NA, and ST developed the algorithms and created scripts for running and evaluating simulation runs. All authors wrote, reviewed, and edited the manuscript. All authors contributed to the article and approved the submitted version.

## Conflict of Interest

The authors declare that the research was conducted in the absence of any commercial or financial relationships that could be construed as a potential conflict of interest.

## Publisher's Note

All claims expressed in this article are solely those of the authors and do not necessarily represent those of their affiliated organizations, or those of the publisher, the editors and the reviewers. Any product that may be evaluated in this article, or claim that may be made by its manufacturer, is not guaranteed or endorsed by the publisher.
