# Supplementary material for: COVIDHunter: COVID-19 Pandemic Wave Prediction and Mitigation via Seasonality Aware Modeling
Source: Front Public Health. 2022 Jun 17;10:877621. doi: 10.3389/fpubh.2022.877621 (PMC9247408; doi:10.3389/fpubh.2022.877621)
Supplement: Supplementary file 1 [file Data_Sheet_1.PDF]

Supplementary Materials

S1 Evaluated datasets

Our experimental evaluation uses a large number of different real datasets, including 1) daily *R* number values, 2) observed daily number of COVID-19 cases, 3) observed daily number of COVID-19 hospitalizations, 4) observed daily number of COVID-19 deaths, 5) number of excess deaths, 6) the estimated strength of mitigation measures as calculated by the Oxford Stringency Index, 7) estimation of COVID-19 statistics as calculated by existing state-of-the-art simulation models, ICL, IHME, LSHTM, and IBZ, from seven different sources as we list below. The raw datasets are provided in the GitHub page of this paper (https://github.com/CMU-SAFARI/COVIDHunter/tree/main/Reproduce-Switzerland-Case-Study-Results) and it can be also obtained from the original sources as we list below:

- Observed COVID-19 statistics (R number values and number of cases, hospitalizations, and deaths)
  - Official reports: https://www.covid19.admin.ch/en/overview
  - Smoothed data: https://ourworldindata.org/coronavirus/country/switzerland?country=~CHE
- Excess deaths:
  - Information: https://www.bfs.admin.ch/bfs/en/home/statistics/health/state-health/mortality-causes-death.html
  - Direct link: https://www.bfs.admin.ch/bfs/en/home/statistics/health/state-health/mortality-causes-death.assetdetail.12607335.html
- Oxford Stringency Index
  - https://www.bsg.ox.ac.uk/research/research-projects/coronavirus-government-response-tracker#data
- The Harvard CRW:
  - Information: https://projects.iq.harvard.edu/covid19/home
  - Direct link: https://projects.iq.harvard.edu/covid19/global
- Imperial College London (ICL) Model:
  - Information: https://mrc-ide.github.io/global-lmic-reports/
  - Direct link: https://github.com/mrc-ide/global-lmic-reports/raw/master/data/2021-04-06v7.csv.zip
- Institute for Health Metrics and Evaluation (IHME) Model:
  - Information: https://mrc-ide.github.io/global-lmic-reports/
  - Direct link: http://www.healthdata.org/covid/data-downloads
- The London School of Hygiene & Tropical Medicine (LSHTM) Model:
  - Information: https://cmmid.github.io/topics/covid19/global_cfr_estimates.html
  - Direct link: https://raw.githubusercontent.com/cmmid/cmmid.github.io/master/topics/covid19/reports/under_reporting_estimates/under_ascertainment_estimates.csv

- The Theoretical Biology Group at ETH Zurich (IBZ) Model:
  - Information: https://ibz-shiny.ethz.ch/covid-19-re-international/
  - Direct link: https://github.com/covid-19-Re/dailyRe-Data

S2 COVIDHunter Prediction Run on 19 April 2021

We provide a comprehensive analysis of the COVID-19 statistics provided by our COVIDHuntter model, ICL (1), IHME (2), IBZ (3), and LSHTM (4) from the beginning of the COVID-19 outbreak (February 2020) until 19 April 2021. We then provide a prediction run for the period from 19 April 2021 until 1 June 2021, which was carried out on 19 April 2021.

S2.1 Observed and predicted R number of SARS-CoV-2

We calculate the predicted R number using our model (**Equation 1**) and compare it to the observed official R number and the R number of two state-of-the-art models, ICL and IBZ, for the two years of 2020 and 2021. We configure COVIDHunter using the following configurations: 1) CTC as environmental condition approach, 2) certainty rate levels of 50% and 100%, and 3) mitigation coefficient values of 0.35 and 0.7. All our scripts are provided on our GitHub page. We consider the mean R number provided by the ICL model. We consider the median R number calculated by the IBZ model based on the observed number of hospitalized patients. IBZ provides the predicted (after 9 April 2021) R number as the mean of the estimates from the last 7 days.

Based on **Figure S1**, we make three key observations. 1) COVIDHunter predicts the changes in R number much (4-13 days) earlier than that predicted by ICL model, which leads to a more accurate prediction. The R number calculated by COVIDHunter (with a certainty rate level of 50%) before 19 April 2021 is on average 1.1× more than that provided by ICL model, IBZ model, and the observed official R number. Using a certainty rate level of 100%, COVIDHunter predicts the R number to be close in value to the observed R number. The R numbers calculated by IBZ model and official authority (observed) are normally not provided for the last two weeks (3)(5). 2) Our model predicts that the current R number is still higher than 1 (1.215 and 1.099 using certainty rate levels of 50% and 100%, respectively) during April 2021. This indicates that the spread of the SARS-CoV-2 virus is still active and it causes an exponential increase in the number of new cases. 3) Our model predicts that if the mitigation measures that are applied nationwide in Switzerland are tightened (*M(t)* increases from 0.55 to 0.7) for only 30 days (19 April to 19 May 2021), then the R number decreases by at least 1.75× (from 1.215 to 0.691). However, if the mitigation measures are relaxed (*M(t)* drops from 0.55 to 0.35) for only 30 days (19 April to 19 May 2021), then the R number increases by at least 1.23× (from 1.215 to 1.497).

We conclude that COVIDHunter's estimation of the R number is more accurate than that calculated by the ICL and IBZ models, as validated by the currently observed R number.

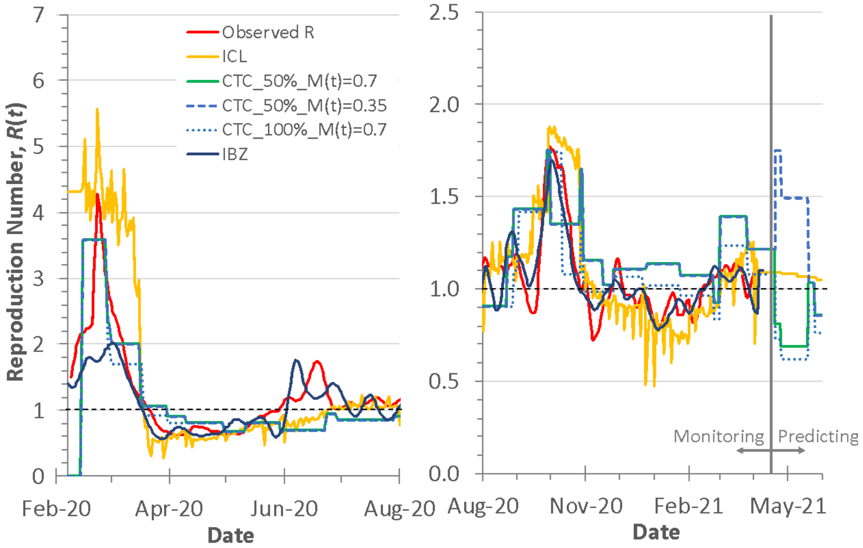

Figure S1. Observed and predicted reproduction number, $R(t)$, for the two years of 2020 and 2021. We use CTC environmental condition approach, certainty rate levels of 50% and 100%, and mitigation coefficient values of 0.35 and 0.7 for COVIDHunter. We compare COVIDHunter's predicted R number to the observed R number and two state-of-the-art models, ICL and IBZ. The horizontal dashed line represents $R(t)$ =1.0.

S2.2 Evaluating the mitigation measures

We evaluate the mitigation coefficient, $M(t)$, which represents the mitigation measures applied (or to be applied) in Switzerland from January 2020 to June 2021. We use two different environmental condition approaches, CRW and CTC. We assume two certainty rate levels of 50% and 100% to account for uncertainty in the observed number of cases. We use five mitigation coefficients, $M(t)$, values of 0.35, 0.4, 0.5, 0.6, and 0.7 for each configuration of COVIDHunter during 19 April to 19 May 2021. We compare the evaluated mitigation measures to that evaluated by the Oxford Stringency Index, as we provide in **Figure S2**. We also evaluate the mitigation coefficient when we ignore the effect of environmental changes (i.e., by setting C_e =1 in **Equation 1**), while maintaining the same number of COVID-19 cases that provided with a certainty rate level of 50%.

Based on **Figure S2**, we make four key observations. 1) Excluding the effect of environmental changes from the COVIDHunter model, by setting C_e=1 in **Equation 1**, leads to an inaccurate evaluation of the mitigation measures. For example, during the summer of 2020 (between the two major waves of 2020), COVIDHunter (*WithoutCTC_50%*) evaluates the mitigation coefficient to be as high as 0.6. This means that the mitigation measures (*only* mandatory wearing masks on public transport) applied during the summer of 2020 are *only* 14% more relaxed compared to the mitigation measures (e.g., closure of schools, restaurants, and borders, ban on small and large events) applied during the first wave, which is implausible. This

highlights the importance of considering the effect of external environmental changes on simulating the spread of COVID-19. Unfortunately, environmental change effects are *not* considered by *any* of the IBZ, LSHTM, ICL, and IHME models, which we believe is a serious shortcoming of these prior models. 2) A drop by 3-30% (as we observe during the mid of November 2020 and the end of August 2020, respectively) in the strength of the mitigation measures for a certain period of time (10 to 20 days) is enough to double the predicted number of COVID-19 cases. 3) We evaluate the strength of the mitigation measures applied in Switzerland to be usually (65% of the time) up to 80% to 131% higher than that provided by the Oxford Stringency Index. 4) The strength of the mitigation measures has changed 11 times and 2 times during the years of 2020 and 2021, respectively, each of which is maintained for at least 9 days and at most 66 days (32 days on average).

We conclude that considering the effect of environmental changes (e.g., daytime temperature) on the spread of COVID-19 improves simulation outcomes and provides an accurate evaluation of the strength of the past and current mitigation measures.

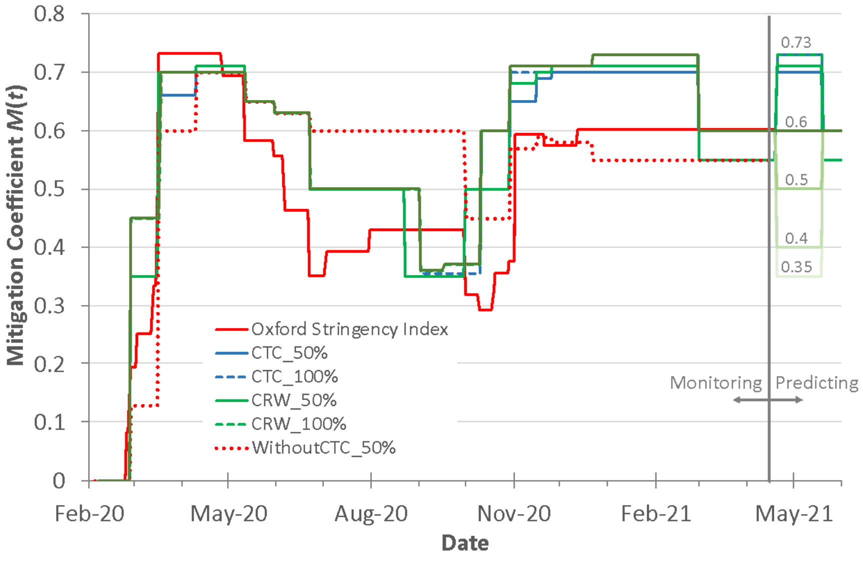

Figure S2. Predicted strength of the mitigation measures (mitigation coefficient, $M(t)$) applied in Switzerland from January 2020 to May 2021 provided by Oxford Stringency Index and COVIDHunter. We use two different environmental condition approaches, CRW and CTC. We assume two certainty rate levels of 50% and 100%. We use five mitigation $M(t)$ values of 0.35, 0.4, 0.5, 0.6, and 0.7 for each configuration of our model during 19 April to 19 May 2021. The plot called WithoutCTC_50% represents the evaluation of the current mitigation measures while ignoring the effect of environmental changes.

S2.3 Evaluating the effect of different mitigation coefficient values on COVIDHunter's predicted number of cases, hospitalizations, and deaths

Using COVIDHunter, we predict the number of COVID-19 cases, hospitalizations, and deaths from 19 April to 19 May 2021. We show the maximum and the average daily number of COVID-19 cases, hospitalizations, and deaths from 19 April to 19 May 2021 in **Figures S3 and S4**, respectively. We use two environmental condition approaches, CRW and CTC, with a certainty rate level of 50%. We assume five mitigation coefficient, $M(t)$, values of 0.35, 0.4, 0.5, 0.6, and 0.7 for each configuration of COVIDHunter from 19 April to 19 May 2021. This range of mitigation coefficient values covers the lowest (i.e., $M(t)$=0.35) and the highest (i.e., $M(t)$=0.7) strengths of mitigation measures that have been applied during the year 2020.

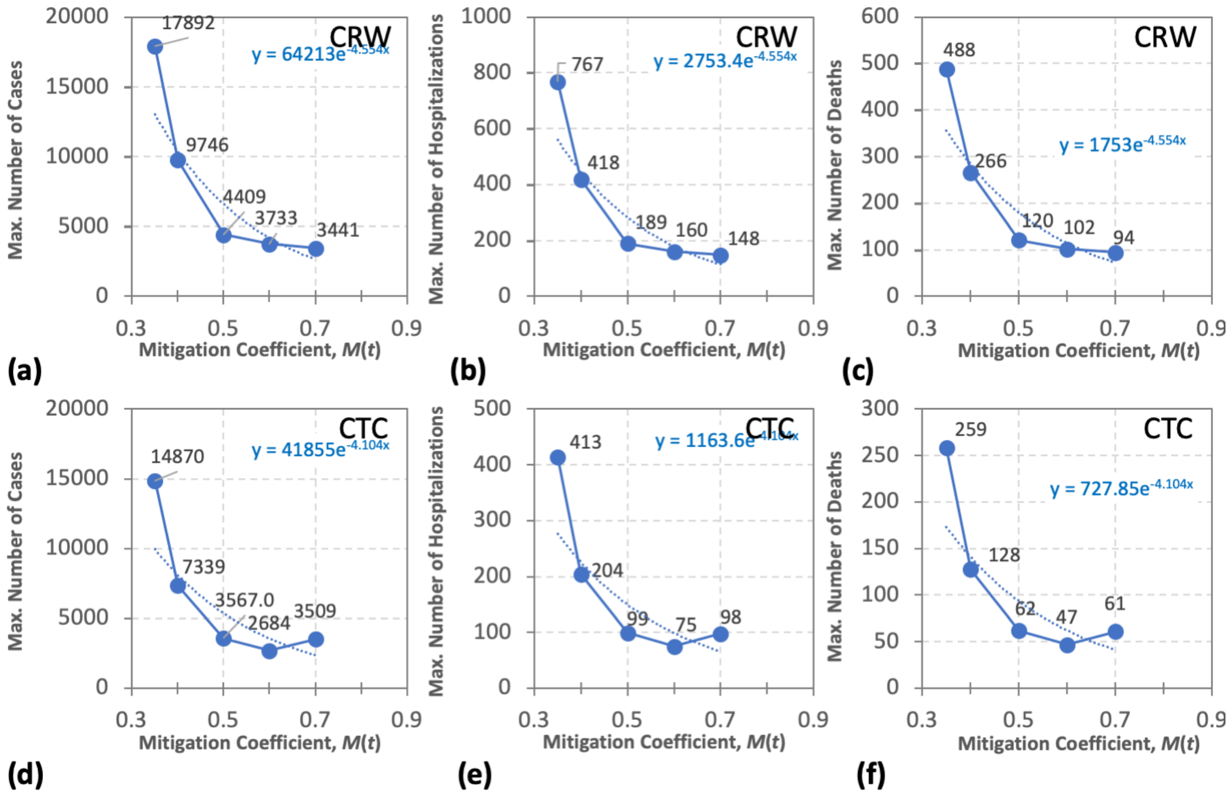

Figure S3: The maximum daily number of COVID-19 cases, hospitalizations, and deaths as predicted by COVIDHunter from 19 April to 19 May 2021. We use five mitigation coefficient, $M(t)$, values of 0.35, 0.4, 0.5, 0.6, and 0.7 for each configuration of our model from 19 April to 19 May 2021. We use two different environmental condition approaches, CRW (a)-(c) and CTC (d)-(f) with a certainty rate level of 50%. The dashed line represents an exponential model fit to data.

Based on **Figures S3 and S4**, we make three key observations. 1) COVIDHunter predicts that the maximum daily number of COVID-19 cases, hospitalizations, and deaths from 19 April to 19 May 2021 would be 3441, 148, and 93, respectively, using CRW and $M(t)$=0.7, as

we show in **Figure S3(a-c)**. Using our environmental condition approach, CTC, and *M*(*t*)=0.7, the maximum daily number of COVID-19 cases, hospitalizations, and deaths from 19 April to 19 May 2021 would be 3509, 98, and 61, respectively, as we show in **Figure S3(d-f)**. 2) Relaxing the mitigation measures (M is changed from 0.55 to 0.35) exponentially increases the maximum daily number of cases, hospitalizations, and deaths by 5.1×, reaching up to 17892, 767, and 488, respectively, as predicted by COVIDHunter with the CRW approach (**Figure S3(a-c)**). Using the CTC approach and *M*(*t*)=0.35, COVIDHunter predicts an exponential increase in the maximum daily number of cases, hospitalizations, and deaths by only 4.13×, as we show in **Figure S3(a-c)**. This is expected as the CTC approach considers only the drop in temperature rather than the average effect of many environmental conditions as the CRW approach does. 3) Relaxing the mitigation measures (*M*(*t*) is changed from 0.55 to 0.35) causes the daily number of cases, hospitalizations, and deaths to exponentially increase by an average of 2.8× and 2.5× from 19 April to 19 May 2021 using CRW and CTC environmental approaches, respectively, as we show in **Figure S4**.

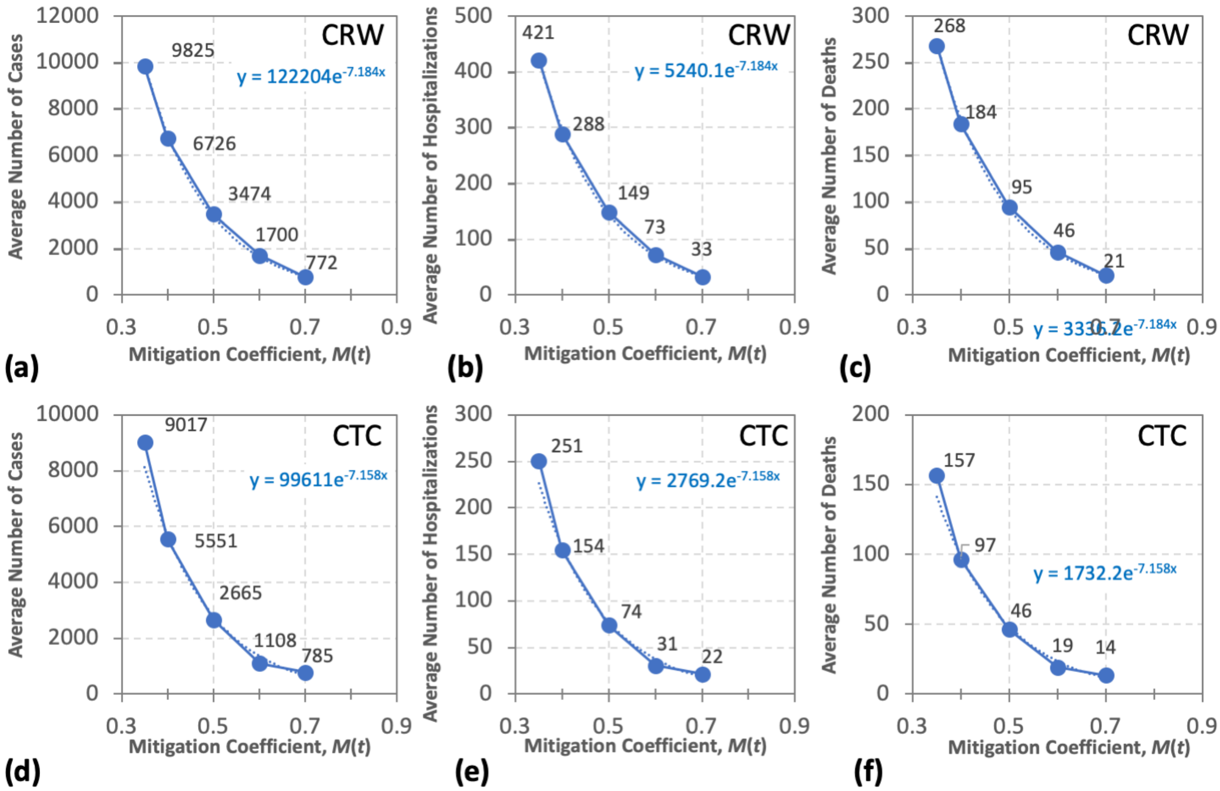

Figure S4: The average daily number of COVID-19 cases, hospitalizations, and deaths as predicted by COVIDHunter from 19 April to 19 May 2021. We use five mitigation coefficient, $M(t)$, values of 0.35, 0.4, 0.5, 0.6, and 0.7 for each configuration of our model from 19 April to 19 May 2021. We use two different environmental condition approaches, CRW (a)-(c) and CTC (d)-(f) with a certainty rate level of 50%. The dashed line represents an exponential model fit to data.

We conclude that COVIDHunter provides a flexible evaluation of the effect of different strengths of the past and current mitigation measures on the number of COVID-19 cases, hospitalizations, and deaths. COVIDHunter evaluates the applied mitigation measures with high flexibility in configuring the environmental coefficient and mitigation coefficient, which helps society and decision-makers to accurately review the current situation and estimate future impact of decisions.

S2.4 Evaluating the predicted number of COVID-19 cases

We evaluate COVIDHunter's *predicted* daily number of COVID-19 cases in Switzerland. We compare the predicted numbers by our model to the observed numbers and those provided by three state-of-the-art models (ICL, IHME, and LSHTM), as shown in **Figure S5**. We calculate the observed number of cases as the expected number of cases with a certainty rate level of 100%. We use three default configurations for the prediction of the ICL model: 1) strengthening

mitigation measures by 50%, 2) maintaining the same mitigation measures, and 3) relaxing mitigation measures by 50% which we refer to as ICL+50%, ICL, and ICL-50%, respectively, in **Figures S5**, **S6**, and **S7**. We use the mean numbers reported by the IHME model that represent the most relaxed mitigation measures, called "no vaccine" by the IHME model. We use the median numbers reported by the LSHTM model.

Based on **Figure S5**, we make four key observations. 1) Our model predicts that the number of COVID-19 cases reduces significantly (less than 50 daily cases) within May 2021 if the mitigation measures that are applied nationwide in Switzerland are tightened (*M(t)* increases from 0.55 to 0.7) for at least 30 days. If the authority decides to relax the mitigation measures to the lowest strength that has been applied during the year 2020 (i.e., $M(t) = 0.35$), then the daily expected number of cases increases by an average of $5.1\times$ and $4.13\times$ (up to 17,892 daily cases) using the CRW and CTC environmental approaches, respectively. 2) COVIDHunter (CTC_100%_M(t)=0.7) predicts the number of COVID-19 cases to be equivalent to that predicted by the IHME model during the second wave with a certainty rate level of 100%. However, during the first wave, the prediction of the IHME model is 3.8× less than the expected number of cases using a certainty rate level of 100%. This means that, unlike our model, the IHME model considers the laboratory-confirmed cases during the first wave to be as if the tests are done at a population-scale, which is very likely incorrect. This is in line with a recent study that demonstrates the high inaccuracy of the IHME model. 3) Overall, our model predicts up to $7.9\times$ and $6.4\times$ (on average $1.9\times$ and $2.1\times$) smaller number of COVID-19 cases than that predicted by ICL model using CTC and CRW approaches, respectively, and a certainty rate of 50%. This suggests that the multiplicative relationship between the confirmed number of cases and the true number of cases can be represented by a certainty rate of 22% to 33%, which our model can easily account for. 4) The number of COVID-19 cases estimated by the LSHTM model during the first wave is 1) on average 24% less than that estimated by COVIDHunter and 2) 10 days late from that predicted by COVIDHunter, IHME, and ICL. The prediction of the LSHTM model during the second wave is not available by the model's pre-computed projections.

We conclude that COVIDHunter provides a more accurate estimation of the number of COVID-19 cases, compared to IHME (which provides inaccurate estimation during the first wave) and ICL (which provides over-estimation), with complete control over the certainty rate level, mitigation measures, and environmental conditions. Unlike LSHTM, COVIDHunter also ensures no prediction delay.

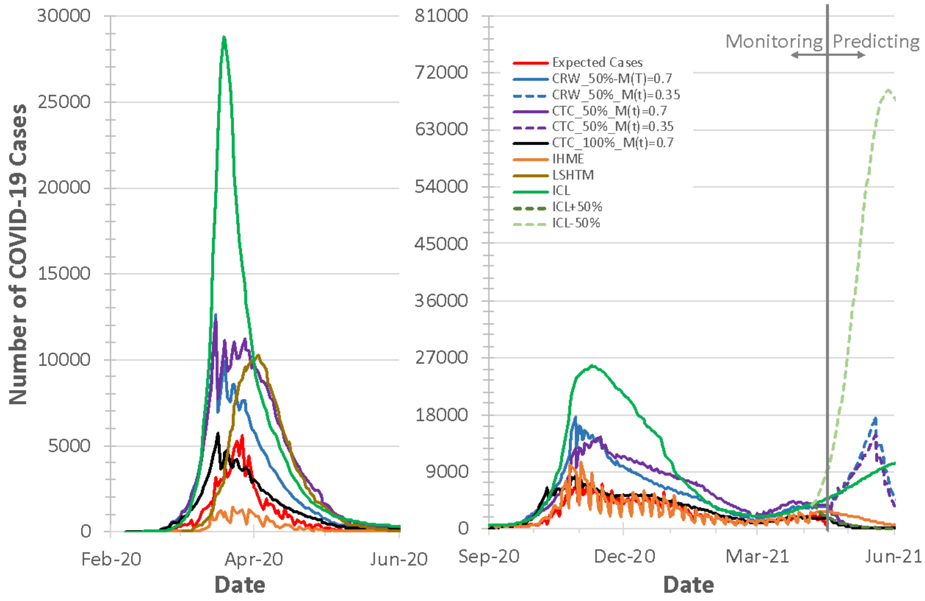

Figure S5. Observed and predicted number of COVID-19 cases by our model and other three state-of-the-art models. We use two different environmental condition approaches, CRW and CTC with two certainty rate levels of 50% and 100%. We use two mitigation coefficient, $M(t)$, values of 0.35 and 0.7 for each configuration of our model from 19 April to 19 May 2021.

S2.5 Evaluating the predicted number of COVID-19 hospitalizations

We evaluate COVIDHunter's *predicted* daily number of COVID-19 hospitalizations in **Figure S6**. We use the observed official number of hospitalizations as is. Using the number of cases calculated with **Equation 2**, we find X (hospitalizations-to-cases ratio) to be 4.288% and 2.780%, using CRW and CTC, respectively, during the second wave.

We make five key observations based on **Figure S6**. 1) COVIDHunter (CRW_50%_M(t)=0.7) with a certainty rate level of 50% predicts on average $5.33\times$ smaller number of COVID-19 hospitalizations than that calculated by the IHME model. 2) The ICL model predicts the number of hospitalizations to be similar to that predicted by COVIDHunter (CTC_50%_M(t)=0.7) during the first and the second waves. This suggests that both the ICL model and COVIDHunter (CTC_50%_M(t)=0.7) consider that the actual number of COVID-19 hospitalizations is twice the observed number of COVID-19 hospitalizations. 3) COVIDHunter with a certainty rate level of 100% predicts the number of cases to perfectly fit the curve of the observed number of hospitalizations, reaching up to 231 hospitalized patients a day. 4) Our model predicts that the number of COVID-19 hospitalizations reduces significantly (less than 5 daily hospitalized patients) within May 2021 if the mitigation measures that are applied nationwide in Switzerland are tightened ($M(t)$ increases from 0.55 to 0.7) for at least 30 days. This is in line with what the ICL model (ICL+50%) predicts when ICL model is configured to strengthen the mitigation measures by 50%. If the authority decides to relax the mitigation

measures to the lowest strength that has been applied during the year 2020 ($M(t)$ drops from 0.55 to 0.35), then the daily expected number of hospitalizations *exponentially* increases by an average of $5.1\times$ and $4.13\times$, becoming as high as the peak of the second wave (up to 767 daily hospitalized patients), using the CRW and CTC environmental approaches, respectively. ICL model predicts the situation to be worst, showing $2\times$ and $3.74\times$ higher number of hospitalizations than COVIDHunter CRW_50%_M(t)=0.35 and CRW_50%_M(t)=0.35, respectively, when ICL model is configured to 50% relaxation in the mitigation measures. 5) The use of the CTC approach for determining the environmental coefficient value yields a slightly different number (on average $1.7\times$ less) of hospitalizations compared to that provided by the use of the CRW approach. This is expected as the CTC approach considers only the monthly average change in temperature, whereas the CRW approach considers the daily change in *several* environmental conditions.

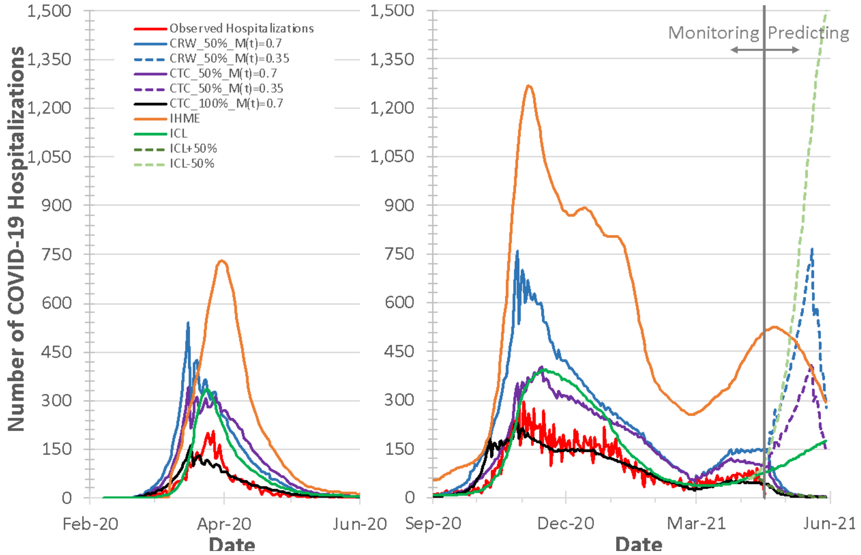

Figure S6. Observed and predicted number of COVID-19 hospitalizations. We use two different environmental condition approaches, CRW and CTC with two certainty rate levels of 50% and 100%. We use two mitigation coefficient values, $M(t)$, of 0.35 and 0.7 for each configuration of our model from 19 April to 19 May 2021.

We conclude that 1) unlike the IBZ and LSHTM models, COVIDHunter is able to predict the number of hospitalizations and 2) COVIDHunter provides more accurate estimation of the number of hospitalizations compared to that calculated by ICL (which provides overestimation) and IHME (which provides late estimation). COVIDHunter predicts the number of COVID-19 hospitalizations in a simple, convenient and flexible way that requires calculating only the daily number of cases and the hospitalization-to-cases ratio, C_X.

S2.6 Evaluating the predicted number of COVID-19 deaths

We evaluate COVIDHunter's *predicted* daily number of COVID-19 deaths in **Figure S7** after accounting for the 15-day shift (as we discuss in **Section S3.3**). We calculate the observed number of deaths as the number of excess deaths to account for uncertainty in reporting COVID-19 deaths. Using the number of cases calculated using **Equation 2**, we find Y (deaths-to-cases ratio, using excess death data) to be 2.730% and 1.739%, using CRW and CTC, respectively, during the second wave.

We make three key observations based on **Figure S7**. 1) COVIDHunter with a certainty rate of 100% predicts the number of deaths to perfectly fit the three curves of the observed number of *excess* deaths, ICL deaths, and IHME deaths, reaching up to 144 deaths a day. During the second wave, the ICL curve is shifted (late prediction) by 5-10 days from that of other models. 2) Similar to what we observe for the number of hospitalizations, our model predicts that the number of COVID-19 deaths significantly reduces (reaching up to a single death a day) with stricter mitigation measures ($M(t)$ increases from 0.55 to 0.7) maintained for at least the upcoming 30 days. This is in line with what the IHME model predicts. Relaxing the mitigation measures ($M(t)$ drops from 0.55 to 0.35) *exponentially* increases the death toll by an average of $5.1\times$ and $4.13\times$, reaching up to 488 new daily deaths, as predicted by COVIDHunter using CRW and CTC environmental condition approaches, respectively. COVIDHunter's prediction (CRW_50%_M(t)=0.35) is in line with what ICL model predicts, when ICL model is configured as 50% relaxation in the mitigation measures. 3) During the first wave, the use of a certainty rate of 50% provides $3\times$ and $2.7\times$ ($2.6\times$ and $1.7\times$ during the second wave) higher number of deaths compared to that provided by ICL and IHME models, when COVIDHunter uses CRW and CTC environmental condition approaches, respectively.

We conclude that 1) unlike the IBZ and LSHTM models, COVIDHunter is able to predict the number of deaths, 2) COVIDHunter predicts the number of deaths to be similar to that predicted by the ICL and IHME models. Yet, COVIDHunter provides a more accurate estimation of other COVID-19 statistics (R, number of cases and hospitalizations) compared to ICL and IHME, as we comprehensively evaluate in the previous sections, and 3) COVIDHunter requires calculating only the daily number of cases and the deaths-to-cases ratio, C_Y, to predict the daily number of deaths.

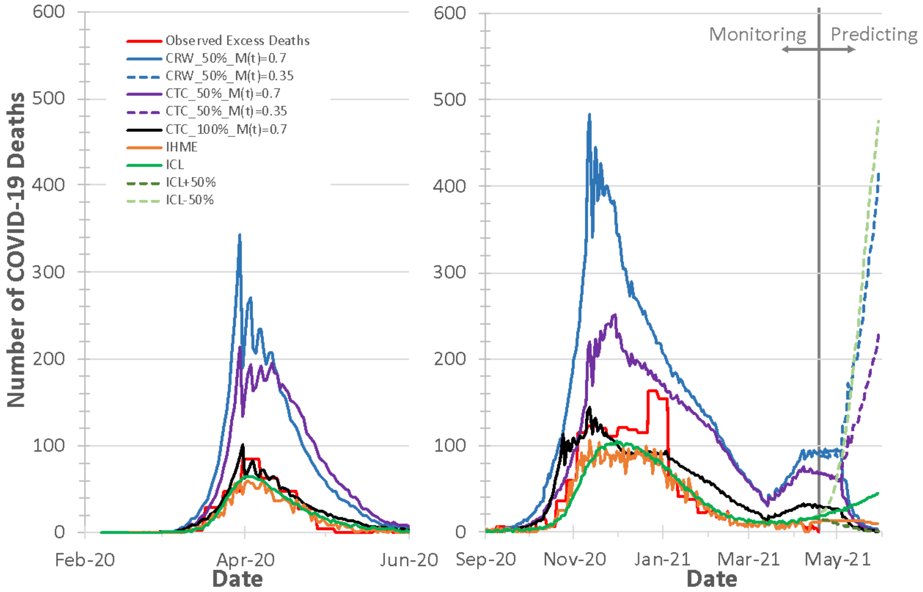

Figure S7. Observed and predicted number of COVID-19 deaths. We use two different environmental condition approaches, CRW and CTC with two certainty rate levels of 50% and 100%. We use two mitigation coefficient values, $M(t)$, of 0.35 and 0.7 for each configuration of our model from 19 April to 19 May 2021.

S2.7 Evaluating the effect of different vaccination rates

We evaluate the effect of different vaccination rates of 0, 0.1, 0.28, 0.4, and 0.5 per day on the reproduction number and the daily number of COVID-19 cases in **Figure S8**. We set the first day of vaccination availability in Switzerland as 28 February 2021 based on governmental data (https://www.covid19.admin.ch/en/overview). We choose May 2021 for our evaluation as it precedes the introduction of the Delta variant in the population of Switzerland and the strength of the mitigation measures remains the same throughout the entire month. This helps us to isolate/reduce potential factors (except the number of vaccinated persons) that can affect the reproduction number and the number of COVID-19 cases. Based on Figure S8, we make three key observations. 1) For each 0.1 rise in the vaccination rate per day, there is on average a 0.07 decrease in the reproduction number, as shown in **Figure S8(a)**. 2) For each 0.1 rise in the vaccination rate per day, there is an exponential decrease in the number of COVID-19 cases, as shown in **Figure S8(b)**.

We conclude that the vaccination rate per day is a key factor that directly affects the average number of new infections caused by each infected person at a given point in time and thus it affects the number of cases.

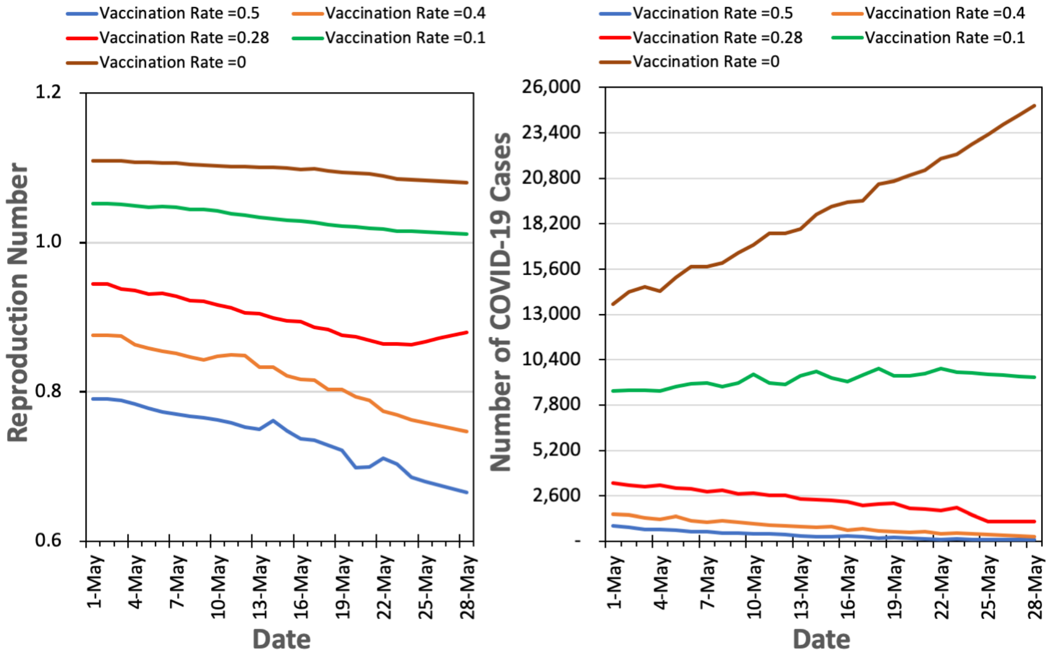

(a) (b)

Figure S8. (a) The reproduction number and (b) the number of COVID-19 cases calculated by COVIDHunter using different vaccination rates per day during May 2021. We use the CTC environmental condition approach with a certainty rate level of 50%.

References

1. Flaxman S, Mishra S, Gandy A, Unwin HJT, Mellan TA, Coupland H, Whittaker C, Zhu H, Berah T, Eaton JW, et al. Estimating the effects of non-pharmaceutical interventions on COVID-19 in Europe. *Nature* (2020) **584**:257–261.
2. IHME COVID-19 Forecasting Team. Modeling COVID-19 scenarios for the United States. *Nat Med* (2021) **27**:94–105.
3. Huisman JS, Scire J, Angst DC, Li J, Neher RA, Maathuis MH, Bonhoeffer S, Stadler T. Estimation and worldwide monitoring of the effective reproductive number of SARS-CoV-2. *medrxiv* (2021)2020–2011.
4. Russell TW, Golding N, Hellewell J, Abbott S, Wright L, Pearson CAB, van Zandvoort K, Jarvis CI, Gibbs H, Liu Y, et al. Reconstructing the early global dynamics of under-ascertained COVID-19 cases and infections. *BMC Med* (2020) **18**:332.
5. COVID-19 Switzerland. https://www.covid19.admin.ch/en/overview [Accessed November 17, 2021]
